# Effects of Betaine on Performance, Blood Biochemistry, Nutrient Utilization and Gut Health in Coccidia‐Infected Broilers

**DOI:** 10.1002/vms3.70779

**Published:** 2026-01-18

**Authors:** Abdul Hafeez, Usama Saleem, Shabana Naz, Rifat Ullah Khan, Muhammad Israr, Ala Abudabos, Ibrahim A. Alhidary

**Affiliations:** ^1^ Department of Poultry Science Faculty of Animal Husbandry and Veterinary Sciences The University of Agriculture Peshawar Pakistan; ^2^ Department of Zoology Government College University Faisalabad Pakistan; ^3^ Physiology Lab College of Veterinary Sciences Faculty of Animal Husbandry and Veterinary Sciences The University of Agriculture Peshawar Pakistan; ^4^ Task Health Care Limited Ilford London, UK United Kingdom; ^5^ Department of Food and Animal Sciences College of Agriculture, Tennessee State University Nashville Tennessee USA; ^6^ Department of Animal Production College of Food and Agriculture Science, King Saud University Riyadh Saudi Arabia

**Keywords:** betaine, coccidiosis, growth performance, lesion score, nutrients digestibility

## Abstract

The objective of this study was to investigate the ameliorative effect of betaine on the performance, blood biochemistry, intestinal lesion score and nutrient utilization of broiler chickens exposed to coccidian challenge. A total of 600 one‐day‐old broiler chicks (Ross 708) were randomly assigned to five groups (six replicates). The negative control group (NC) remained unchallenged and received non‐supplemented feed, while the positive control group (PC) received non‐supplemented feed and was challenged with oocysts. The other three groups (B2, B4 and B6) were challenged with oocysts and received feed supplemented with 200, 400 and 600 mg/kg betaine, respectively. Results indicated a significant decrease in growth performance, nutrient digestibility and altered blood lipid profiles in the PC group, with the most significant restoration observed in the B6 group. Caecal lesion scores were also notably restored in the B4 and B6 groups among the infected broilers. In conclusion, betaine supplementation at a rate of 600 mg/kg demonstrated improvements in growth performance, nutrient digestibility, blood biochemistry and caecal lesion scores in broilers experimentally exposed to coccidiosis. However, the findings cannot be considered 100% conclusive, as variations in breed, management practices and infection severity may influence outcomes. The major limitation of this study is that it was conducted under controlled experimental conditions, which may not fully reflect field circumstances. Future research should explore different genetic strains, longer trial durations and combinations of betaine with other anticoccidial alternatives to better validate and extend these results.

## Graphical abstract

Betaine supplementation improved growth performance, nutrient digestibility, blood lipid profiles and caecal lesion scores in broilers experimentally challenged with coccidiosis, with the greatest benefits observed at 600 mg/kg, highlighting its potential as a supportive dietary strategy against coccidial infection.

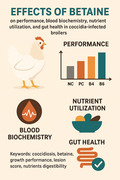

## 1 Introduction

Coccidiosis, an enteric protozoan parasitic disease in poultry, is associated with substantial economic losses primarily attributed to severe diarrhoea and elevated mortality rates (Rashid and Shnawa 2024; Batool et al. 2025). Most of these economic burdens arise from diminished performance, with the remainder stemming from prophylactic and treatment expenses (Abbas et al. 2025; Nasir et al. 2023). Coccidial infection in broilers induces epithelial cell damage, diarrhoea and intestinal osmotic stress (Khan et al. 2023), subsequently leading to nutrient malabsorption (Saeeda et al. 2023; Almahallawi et al. 2024). Decreases in nutrient and energy digestibility have been documented in birds exposed to coccidia, and the extent of these responses is influenced by diet composition as well as the type and quantity of *Eimeria* species introduced in the challenge model (Ishaq et al. 2022).

Betaine is a naturally occurring compound first isolated from sugar beet (*Beta vulgaris*) and widely present in plants, microorganisms and animal tissues (Ghasemi and Nari 2020). Chemically, it is a trimethyl derivative of the amino acid glycine, often referred to as trimethylglycine (Chand et al. 2017). Its unique zwitterionic structure enables it to function both as a methyl donor and as an osmolyte, making it particularly valuable in poultry nutrition (Saeed et al. 2017). Dietary supplementation with betaine has been reported to positively influence growth performance, metabolism and immunity (Chand et al. 2017; Ghasemi and Nari 2020). As a lipotropic agent, betaine reduces hepatic lipid deposition and enhances lipid turnover in the body, proving useful in diets with a low crude protein and high metabolizable energy‐to‐protein ratio. In addition, its osmoregulatory effect supports immune, cardiovascular, nervous and renal functions (Sakomura et al. 2013). These properties enable betaine to alleviate heat stress (Saeed et al. 2017), protect intestinal enzymes and improve nutrient digestibility (Chand et al. 2017).

Despite these established benefits, limited studies have directly examined the role of betaine in mitigating intestinal damage and nutrient malabsorption specifically under *Eimeria*‐induced coccidiosis in broilers. Most prior work has focused on its effects under nutritional stress or heat stress, leaving a critical gap in understanding its potential as an alternative or adjunct to conventional anticoccidials.

Therefore, this study was designed to address this gap by investigating whether dietary betaine supplementation at different inclusion levels could alleviate intestinal lesions, improve nutrient digestibility and restore growth performance and blood biochemistry in broilers experimentally challenged with *Eimeria* spp.

## 2 Materials and Methods

### 2.1 Birds and Treatments

A total of 600 one‐day‐old broiler chicks (Ross 708) were acquired, each weighing approximately 42 ± 2 g. The chicks were subjected to a lighting program of 23 h of light and 1 h of darkness. They were housed in pens with dried wood shavings, and throughout the study, access to feed and water was provided ad libitum. The broiler chicks were fed a corn‐soybean meal basal diet, free of coccidiostats, which met the recommended requirements outlined in the Ross catalogue for both the starter and finisher phases. The broiler chicks were randomly divided into five groups, each consisting of six replicates and 20 birds per group. The first group served as the negative control (NC), receiving non‐supplemented feed and remaining unchallenged with oocysts. The second group, positive control (PC), received non‐supplemented feed but was challenged with oocysts. The remaining three groups, B2, B4 and B6, were challenged with oocysts and received feed supplemented with 200, 400 and 600 mg/kg betaine, respectively. Betaine (anhydrous betaine, ≥ 99% purity) was procured from Sigma‐Aldrich, St. Louis, MO, USA]. On Day 21, all groups, except the NC, were subjected to a challenge with a mixture of *Eimeria* species, involving the oral inoculation of a 1.5 mL solution containing sporulated oocysts (2 × 10^5^ oocysts). The *Eimeria* species composition included *Eimeria necatrix* (10%), *E. acervulina* (11%), *E. maxima* (9%), and *E. tenella* (70%). The solution also contained 96% natural betaine from Marlborough, UK. In addition, the diets were formulated with chromium dioxide (0.3%) as an inert marker, and the pellets were produced at 70°C. The diets were provided ad libitum (Table 1), and water access was unrestricted throughout the entire trial.

**TABLE 1:** Composition of experimental diets from 1–42 days.

	1–21 days	22–45 days
Corn	60.00	61.00
Soybean meal (46.8%)	30.00	28.0
Soybean oil	3.50	4.50
Corn gluten meal	2.00	2.70
Limestone	1.30	1.50
Dicalcium phosphate	2.00	1.50
Salt	0.10	0.10
Premix	0.50	0.50
L‐Methionine	0.13	0.19
Calculated nutrient contents		
Crude protein (%)	22.0	19.50
Metabolizable energy (MJ/kg)	12.30	12.50
Lysine (%)	1.25	1.10
Calcium (%)	1.00	0.95
Methionine (%)	0.45	0.40
Available phosphorus (%)	0.4	0.36

*Note*: Premix was provided the following for per kg of diet: vitamin A (transretinyl acetate), 10,000 U; vitamin D3 (cholecalciferol), 3000 U; vitamin E (allrac‐ a‐tocopherol acetate), 30 mg; menadione, 1.3 mg; thiamine, 2.2 mg; riboflavin, 8 mg; nicotinamide, 40 mg; choline chloride, 600 mg; calcium pantothenate, 10 mg; pyridoxine HCl, 4 mg; biotin, 0.04 mg; folic acid, 1 mg; vitamin B12 (cobalamin), 0.013 mg; Fe (from ferrous sulphate), 80 mg; Cu (from copper sulphate), 8 mg; Mn (from manganese sulphate), 110 mg; Zn (from zinc oxide), 65 mg; I (from calcium iodate), 1.1 mg; and Se (from sodium selenite), 0.3 mg.

### 2.2 Growth Performance

Feed intake (FI) was calculated as the difference between the offered and refused feed. Weight gain (WG) of chicks was determined as the difference in bird weight at the end and start of each week. The feed conversion ratio (FCR) was computed by dividing the total amount of feed consumed by chicks by their WG. Mortality was monitored daily, and any dead birds were recorded. Since no mortality occurred during the trial (despite coccidial challenge), growth performance data were not confounded by mortality effects (Hafeez et al. 2020).

### 2.3 Lesion Score

On Day 7 post‐challenge (Day 21 post‐hatch), two birds were randomly chosen from each replicate cage, euthanized by cervical dislocation, and lesion scoring was performed by a skilled avian veterinarian unaware of treatment allocations. Coccidiosis lesions in the ceca were assessed on a scale of 0–4, following the method outlined by Almahallawi et al. (2024). A score of zero indicated the *absence of gross lesions*, while 4 denoted *extensive haemorrhage or lesions*, depending on the *Eimeria* species involved.

### 2.4 Apparent Total Digestibility

On Day 42, two birds per replicate were selected, weighed, stunned and euthanized by exsanguination. Faecal samples were collected from the cloaca and subsequently freeze‐dried at −20°C for further chemical analysis. The apparent total digestibility (ATD) of nutrients was determined using the formula (Ullah et al. 2022):

$$\begin{array}{c} 100-[(\mathrm{chromium}\;\mathrm{in}\;\mathrm{feed}/\mathrm{chromium}\;\mathrm{in}\;\mathrm{feces})\times \\ (\mathrm{nutrient}\;\mathrm{in}\;\mathrm{feces}/\mathrm{nutrient}\;\mathrm{in}\;\mathrm{feed})\times 100] \end{array}$$

### 2.5 Blood Biochemical Analysis

On the 42nd day of the trial, blood samples were extracted from the wing veins of two birds per treatment and placed in tubes without anticoagulant. The blood samples underwent centrifugation for 12 min at 1300 × *g* and 4°C. Subsequently, sera were collected and stored at −20°C for later analysis (Chand et al. 2018). Triglycerides (TG), total cholesterol (CHOL), LDL, HDL and total protein levels were measured using a commercial kit (Biocheck, UK; Cat. No. BC1117, Cholesterol Cat. No. BC1120, LDL Cat. No. BC1122, HDL Cat. No. BC1124, and Total Protein Cat. No. BC1126).

### 2.6 Cecum Histology

On Day 42, two birds per treatment group were randomly selected and euthanized for histological evaluation of caecal tissues. Segments (approximately 2 cm) from the mid‐jejunum and cecum were excised, gently rinsed in phosphate‐buffered saline (PBS) and fixed in 10% neutral buffered formalin for 48 h. Fixed tissues were dehydrated through a graded ethanol series, cleared in xylene and embedded in paraffin wax. Sections of 5 µm thickness were cut using a rotary microtome and mounted on glass slides. The sections were stained with haematoxylin and eosin (H&E) for general histopathological examination under a light microscope (×40 magnification).

### 2.7 Statistical Analysis

Data were tested for normality and homogeneity of variance using the Shapiro–Wilk and Levene's tests, respectively. One‐way analysis of variance (ANOVA) was performed using SPSS (version 20.0; IBM, Armonk, NY, USA). When ANOVA indicated a significant treatment effect (*p* < 0.05), pairwise comparisons were performed using Tukey's Honestly Significant Difference (HSD) post‐hoc test. Data are presented as mean ± SEM. In tables, means within the same row bearing different superscript letters are significantly different (*p* < 0.05) according to Tukey's HSD.

## 3 Results

### 3.1 Growth Performance

Table 2 illustrates the impact of betaine supplementation in the broiler diet on FI. In the first week, FI exhibited a statistically significant increase (*p* < 0.05) in the B6 treatment compared to the B4 treatment. At Week 2, FI showed a statistically significant increase (*p* < 0.05) in the B6 treatment, B4 treatment and B2 treatment compared to the PC treatment and NC treatment. By Week 3, FI was significantly (*p* < 0.05) higher in the B6 treatment, B4 treatment and NC treatment in comparison to the B2 treatment and PC treatment.

**TABLE 2:** Effect of dietary supplementation of betaine in broilers challenged with coccidiosis on feed intake (g).

Groups	NC	PC	B2	B4	B6	SEM	*p*‐value
Week‐1	118^d^	116^cd^	124^c^	130^b^	135^a^	1.94	<0.001
Week‐2	321^b^	324^b^	334^a^	338^a^	343^a^	2.32	<0.001
Week‐3	529^a^	521^b^	537^bc^	544^ab^	552^a^	2.97	<0.001
Starter (d1–21)	969^d^	961^d^	994^c^	1012^b^	1030^a^	7.01	<0.001
Week‐4	837^a^	762^b^	756^bc^	748^cd^	740^d^	9.33	<0.001
Week‐5	959^a^	925^b^	924^b^	910^c^	905^c^	5.07	<0.001
Week‐6	1074^a^	1011^d^	1024^c^	1038^b^	1072^a^	6.80	<0.001
Finisher (d21–42)	2869^a^	2699^c^	2705^bc^	2696^c^	2717^b^	17.8	<0.001
Overall (d1–42)	3838^a^	3660^d^	3699^c^	3708^c^	3747^b^	16.3	<0.001

*Note*: Means with different superscripts in the same row are significantly different at *p *< 0.05.

Abbreviations: B2, B4 and B6, betaine supplementation at the rate of 200, 400 and 600 mg/kg, NC, negative control; PC, positive control.

During the starter period, FI demonstrated a statistically significant increase (*p* < 0.05) in the B6 treatment compared to the other treatments. At Week 4, FI exhibited a statistically significant increase (*p* < 0.05) in the NC treatment compared to the other treatments. In Week 5, FI was significantly (*p* < 0.05) higher in the NC treatment compared to the other treatments. By Week 6, FI was significantly (*p* < 0.05) higher in the NC treatment and B6 treatment compared to the other treatments. During the finisher period, FI demonstrated a statistically significant increase (*p* < 0.05) in the NC treatment compared to the other treatments. For the overall period, FI was significantly (*p* < 0.05) higher in the NC treatment and significantly (*p* < 0.05) lower in PC treatment of broilers.

Table 3 outlines the influence of betaine inclusion in the broiler diet on WG. In the initial week, WG displayed a statistically significant elevation (*p* < 0.05) in the B6 treatment, while being significantly reduced (*p* < 0.05) in both the NC and PC treatments. In the second week, WG showed a statistically significant increase (*p* < 0.05) in both the B6 and B4 treatments, with a concurrent significant decrease (*p* < 0.05) in the PC and NC treatments. By the third week, WG was significantly higher (*p* < 0.05) in the B6 treatment, while being significantly lower (*p* < 0.05) in both the NC and PC treatments.

**TABLE 3:** Effect of dietary supplementation of betaine in broilers challenged with coccidiosis on weight gain (g).

Groups	NC	PC	B2	B4	B6	SEM	*p*‐value
Week‐1	102^d^	102^d^	111^c^	118^b^	125^a^	2.47	<0.001
Week‐2	252^c^	250^c^	262^b^	274^a^	280^a^	3.25	<0.001
Week‐3	356^c^	352^c^	369^b^	376^b^	387^a^	3.52	<0.001
Starter (d1–21)	709^d^	705^d^	742^c^	768^b^	793^a^	9.09	<0.001
Week‐4	498^a^	418^c^	426^bc^	428^bc^	436^b^	7.82	<0.001
Week‐5	559^a^	495^d^	510^c^	526^b^	532^b^	5.82	<0.001
Week‐6	607^b^	539^e^	570^d^	584^c^	621^a^	7.77	<0.001
Finisher (d21–42)	1665^a^	1452^e^	1507^d^	1537^c^	1590^b^	19.5	<0.001
Overall (d1–42)	2374^a^	2156^d^	2249^c^	2305^b^	2383^a^	22.6	<0.001

*Note*: Means with different superscripts in the same row are significantly different at *p *< 0.05.

Abbreviations: B2, B4 and B6, betaine supplementation at the rate of 200, 400 and 600 mg/kg; NC, negative control; PC, positive control.

During the starter phase, WG exhibited a statistically significant increase (*p* < 0.05) in the B6 treatment and significantly (*p* < 0.05) lower in PC treatment, and NC treatment. At Week 4, WG showed a statistically significant increase (*p* < 0.05) in the NC treatment compared to the rest of the treatment. In Week 5, WG was significantly (*p* < 0.05) higher in the NC treatment and significantly (*p* < 0.05) lower in PC treatment. By Week 6, WG exhibited a statistically significant increase (*p* < 0.05) in the B6 treatment compared to the NC treatment. During the finisher phase, WG demonstrated a statistically significant increase (*p* < 0.05) in the NC treatment and significantly (*p* < 0.05) lower in PC treatment. For the overall period, WG was significantly (*p* < 0.05) higher in both the NC treatment and B6 treatment and significantly (*p* < 0.05) lower in PC treatment.

Table 4 outlines the impact of incorporating betaine into the broiler diet on FCR. In the first week, FCR demonstrated a statistically significant decrease (*p* < 0.05) in the B6 treatment and significantly (*p* < 0.05) low in the NC treatment. During the second week, FCR exhibited a statistically significant reduction (*p* < 0.05) in both the B6 treatment and B4 treatment compared to the PC. By Week 3, FCR was significantly (*p* < 0.05) lower in the B6 treatment compared to the NC treatment.

**TABLE 4:** Effect of dietary supplementation of betaine in broilers challenged with coccidiosis on feed conversion ratio (FCR).

Groups	NC	PC	B2	B4	B6	SEM	*p*‐value
Week‐1	1.16^a^	1.14^ab^	1.12^ab^	1.10^ab^	1.08^b^	0.010	0.035
Week‐2	1.27^ab^	1.29^a^	1.27^ab^	1.23^b^	1.22^b^	0.008	0.005
Week‐3	1.49^a^	1.48^bc^	1.45^bc^	1.45^bc^	1.43^c^	0.007	0.004
Starter (d1–21)	1.36^a^	1.36^a^	1.34^ab^	1.32^bc^	1.30^c^	0.007	<0.001
Week‐4	1.68^c^	1.82^a^	1.78^b^	1.75^b^	1.70^c^	0.014	<0.001
Week‐5	1.71^cd^	1.87^a^	1.81^b^	1.73^c^	1.70^d^	0.017	<0.001
Week‐6	1.77^c^	1.88^a^	1.80^b^	1.78^bc^	1.72^d^	0.014	<0.001
Finisher (d21–42)	1.72^d^	1.86^a^	1.79^b^	1.75^c^	1.71^d^	0.015	<0.001
Overall (d1–42)	1.62^c^	1.70^a^	1.64^b^	1.61^c^	1.57^d^	0.011	<0.001

*Note*: Means with different superscripts in same row are significantly different at *p *< 0.05.

Abbreviations: B2, B4 and B6, betaine supplementation at the rate of 200, 400 and 600 mg/kg; NC, negative control; PC, positive control.

In the starter phase, the FCR showed a statistically significant reduction (*p* < 0.05) in the B6 and B4 treatments, while significantly increasing (*p* < 0.05) in the NC and PC treatments. By Week 4, FCR displayed a statistically significant decrease (*p* < 0.05) in the B6 treatment and a significant increase (*p* < 0.05) in the PC treatment. In Week 5, FCR was significantly lower (*p* < 0.05) in the B6 treatment and significantly higher (*p* < 0.05) in the PC treatment. By Week 6, FCR exhibited a statistically significant decrease (*p* < 0.05) in the B6 treatment and a significant increase (*p* < 0.05) in the PC treatment. During the finisher phase, FCR demonstrated a statistically significant decrease (*p* < 0.05) in both the B6 and NC treatments compared to the PC treatment. For the overall period, FCR was significantly lower (*p* < 0.05) in the B6 treatment compared to the PC treatment.

### 3.2 Feed Digestibility

Table 5 summarizes the impact of incorporating betaine into the broiler diet on the percentage of ATD of nutrients. The ATD% of dry matter (DM) was significantly (*p < *0.05) higher in NC treatment and B6 treatment as compared to B2 treatment and B4 treatment followed by PC treatment. The ATD% of ash was significantly (*p *< 0.05) higher in NC treatment as compared to PC treatment with no differences in B2 treatment, B4 treatment and B6 treatment. The ATD% of crude protein was significantly (*p *< 0.05) higher in B6 treatment and NC treatment as compared to B2 treatment followed by PC treatment, but without any differences with B4 treatment. The ATD% of crude fat was significantly (*p *< 0.05) higher in NC treatment in comparison with B6 treatment followed by B4 treatment, B2 treatment and PC treatment.

**TABLE 5:** Effect of dietary supplementation of betaine in broilers challenged with coccidiosis on apparent total digestibility of nutrients (%).

Groups	NC	PC	B2	B4	B6	SEM	*p*‐value
Dry matter	72.7^a^	52.2^d^	60.5^c^	65.3^bc^	68.3^ab^	1.41	<0.001
Ash	49.3^a^	44.7^b^	45.8^ab^	49.0^ab^	49.2^ab^	0.58	0.012
Crude protein	70.8^a^	60.3^c^	66.3^b^	67.8^ab^	71.5^a^	0.83	<0.001
Crude Fat	79.8^a^	52.8^e^	59.5^d^	67.5^c^	73.5^b^	1.84	<0.001

*Note*: Means with different superscripts in same row are significantly different at *p *< 0.05.

Abbreviations: B2, B4 and B6, betaine supplementation at the rate of 200, 400 and 600 mg/kg; NC, negative control; PC, positive control.

### 3.3 Blood Biochemistry and Caecal Lesion Score

**FIGURE 1:**
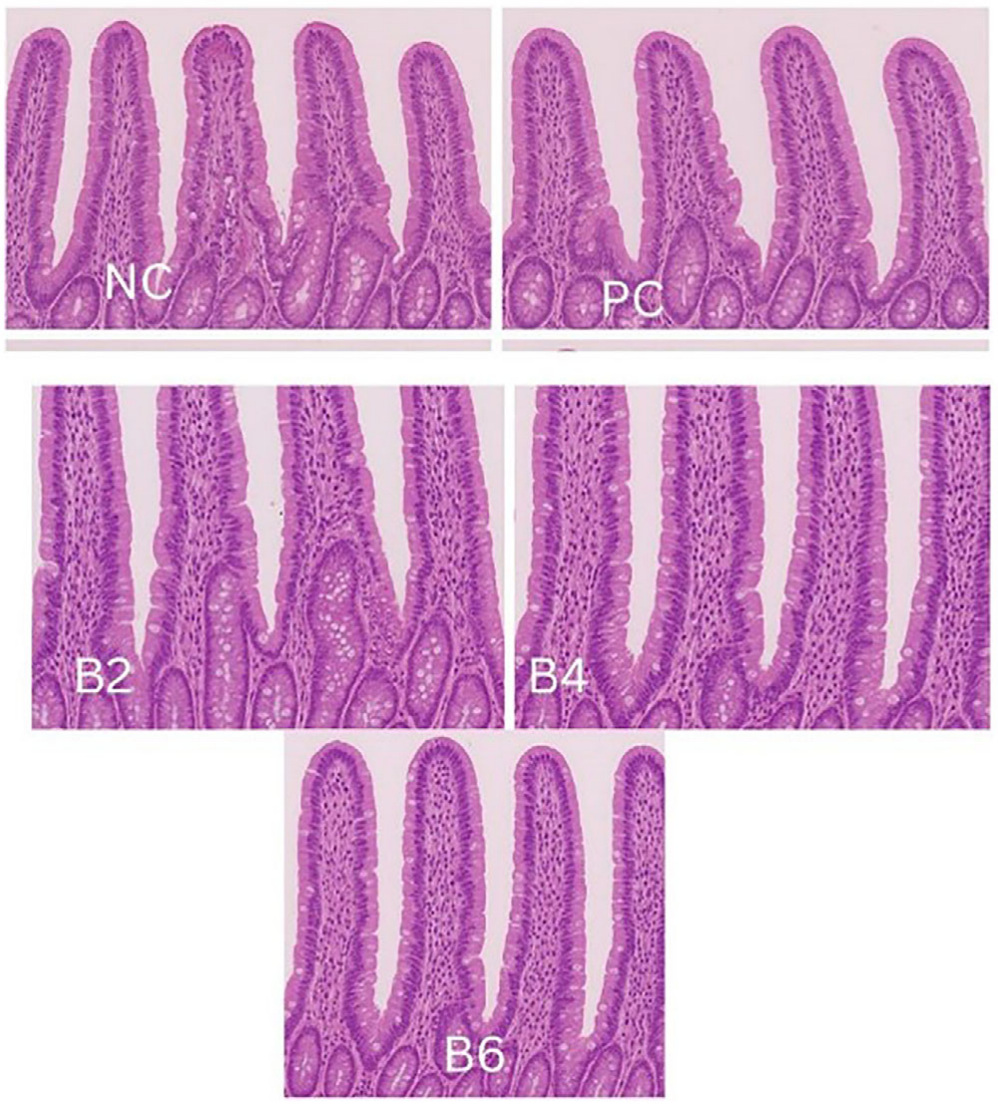
NC showed normal caecal architecture with intact villi and no inflammation. PC exhibited severe necrosis, villus destruction, haemorrhage and heavy inflammatory infiltration. B2 showed moderate villus shortening, crypt hyperplasia and focal necrosis. B4 displayed mild to moderate lesions with improved villi and reduced inflammation. B6 revealed minimal changes, near‐normal villi, and mild inflammatory infiltration.

Table 6 and Figure 1, show the effect of inclusion of betaine in broilers diet on blood lipid profile and lesion score. TGs concentration was significantly (*p *< 0.05) higher in B4 and B6 treatment and significantly (p < 0.05) low in PC treatment. Total CHOL concentration was significantly (*p *< 0.05) higher in B4, B6 and NC treatments and significantly (*p* < 0.05) low in PC treatment. The LDL concentration was significantly (*p *< 0.05) higher in NC treatment in comparison with PC treatment. The HDL concentration was significantly (*p *< 0.05) lower in PC treatment compared to all other treatments. Total protein concentration was not significantly affected (*p* > 0.05) among all treatments. Lesion score reduced significantly (*p* < 0.05) in B4 and B6 compared to the PC treatment. No lesions were observed in the intestine or ceca of the unchallenged treatments.

**TABLE 6:** Effect of dietary supplementation of Betaine in broilers challenged with coccidiosis on Blood Lipid Profile and lesion score in ceca.

Groups	NC	PC	B2	B4	B6	SEM	*p*‐value
Triglycerides (mg/dL)	47.5^cd^	45.0^d^	48.8^bc^	51.0^ab^	53.3^a^	0.589	<0.001
Total cholesterol (mg/dL)	112^ab^	101^c^	110^b^	112^ab^	114^a^	0.923	<0.001
LDL (mg/dL)	49.0^a^	45.5^c^	46.8^ab^	48.2^ab^	47.8^ab^	0.383	0.029
HDL (mg/dL)	53.5^a^	46.7^b^	53.0^a^	53.7^a^	55.5^a^	0.615	<0.001
Total Protein (g/dL)	4.27	4.42	4.55	4.40	4.45	0.034	0.122
Caecal lesion score	0.00^c^	2.1^a^	1.99^a^	1.91^b^	1.90^b^	0.331	0.034

*Note*: Means with different superscripts in same row are significantly different at *p *< 0.05.

Abbreviations: B2, B4 and B6, betaine supplementation at the rate of 200, 400 and 600 mg/kg; NC, negative control; PC, positive control.

## 4 Discussion

### 4.1 Growth Performance

The results indicated that coccidial infection had a negative impact on the growth performance during the grower and finisher periods (Zhang et al. 2024). Growth performance, a crucial metric in the poultry industry, reflects feed utilization and efficiency (Rahman et al. 2017). Similarly, Batool et al. (2025) observed a significant reduction in the growth performance of broiler chicks challenged with *Eimeria* spp. Coccidiosis was found to hinder nutrient digestion and absorption, affecting the overall growth performance in broiler chicks (Ishaq et al. 2022). Previous studies have highlighted a connection between impaired performance due to coccidia challenge and a decrease in absorptive surface area, nutrient malabsorption and inflammation (Hafeez et al. 2023; Nasir et al. 2023). In our study, supplementation with 0.6% betaine effectively restored growth performance in challenged broilers. The observed decrease in WG among infected birds in our study seems to be attributed to both compromised FI and feed utilization. Birds experimentally infected with *Eimeria* spp. has suggested that diminished feed utilization contributes significantly to the decline in WG (Chand et al. 2021; Khan et al. 2023a; S. Khan et al. 2023b). Betaine functions as a methyl group donor (Kar et al. 2025). In conditions where diets are deficient in methionine, the inclusion of betaine serves to compensate for the role of methionine as a methyl donor, leading to improved performance (Alsayeqh and Rao 2023). This ability of betaine to serve as a methyl group donor may explain its positive impact on FCR in birds facing nutritional challenges. Moreover, under challenging conditions such as coccidia infection, there is an increased demand for methyl donors (Kar et al. 2025), highlighting the significance of betaine as a crucial methyl donor under such circumstances.

In this study, coccidia challenge led to notable reductions in the apparent ileal digestibility of DM, ash, crude protein and fat. Coccidiosis‐induced damage to the intestinal lining, particularly the shortening of villi, and decreased activities of digestive enzymes contribute to compromised nutrient digestion and absorption. The observed decline in fat digestibility during coccidia challenge may involve additional mechanisms, such as a reported reduction in bile salt secretion due to mucosal cell damage. Cholecystokinin, responsible for gallbladder contraction and pancreatic enzyme secretion, may be impacted by coccidia challenge. Notably, betaine supplementation demonstrated an improvement in digestibility in birds facing coccidia challenge. Previous research suggests that betaine may influence the crypt‐to‐villus height ratio, providing a potential explanation for the enhanced nutrient digestibility observed with betaine supplementation, as reported by Awad et al. (2022). Betaine, acting as an osmoprotectant, exerts effects on promoting intestinal cell growth, enhancing intestinal cell activity and improving intestinal morphology.

### 4.2 Blood Biochemistry

The current findings indicate that coccidiosis led to decreased serum concentrations of LDL‐C, TG, VLDL‐C, CHOL and HDL‐C. These findings are consistent with prior studies, which noted decreased plasma levels of TGs and elevated CHOL concentrations in broiler chickens infected with coccidia compared to a non‐infected group. Furthermore, Su et al. (2025) documented diminished serum levels of CHOL, TGs and HDL in coccidia‐infected broiler chicks compared to a control group. As demonstrated in the current study, the addition of dietary betaine resulted in a significant increase in serum TG and showed a tendency towards higher CHOL concentration at Day 42 of age. Yusuf et al. (2018) observed an increase in serum CHOL with dietary betaine supplementation (800 mg/kg) in broilers, while serum TG levels remained unchanged. Ghasemi and Nari (2020) reported a higher lipid profile with no change in blood protein in heat‐stressed broilers fed 1 g/kg betaine supplementation. The observed incremental effect of dietary betaine on serum TG in the current study is likely attributed to its role in mobilizing stored fat into the blood vessels for metabolism and oxidation in tissues.

### 4.3 Caecal Lesions

In the current study, caecal lesion score was restored in infected broilers received 400 and 600 mg/kg betaine. The protective impact of betaine in coccidial infection stems from its dual functionality as both an osmolyte and a methyl group donor (Kar et al. 2025). Acting as an osmolyte, betaine helps alleviate the osmotic stress experienced in the intestinal tract during coccidiosis (Alsayeqh and Rao 2023). Kar et al. (2025) found that chicks supplemented with betaine exhibited a significant reduction in the intracellular invasion of *E. tenella* or *E. acervulina* sporozoites in the intestines compared to control chicks. In its role as a methyl donor, betaine is thought to provide additional methyl groups, particularly to damaged tissues, which have higher requirements for these groups than healthy tissues (El‐Ghany and Babazadeh 2022).

## 5 Conclusion

Betaine supplementation at 600 mg/kg effectively mitigated the adverse effects of coccidiosis in broilers, improving growth performance, nutrient digestibility, blood lipid profiles and caecal lesion scores. However, these findings were obtained under controlled experimental conditions, which may not fully represent field circumstances where multiple stressors interact. Moreover, only one genetic line of broilers and a single species of *Eimeria* were tested, limiting the generalizability of the results. Future studies should explore the efficacy of betaine across different broiler strains, ages and production systems, and in combination with other phytogenic or nutritional additives. Long‐term field trials are also needed to validate its potential as a sustainable alternative to conventional anticoccidials.

## Author Contributions

**Abdul Hafeez**: conceptualization. **Usama Saleem**: software; methodology; data curation. **Shabana Naz**: visualization; validation. **Rifat Ullah Khan**: writing – review and editing; writing – original draft. **Muhammad Israr**: writing – review and editing; writing – original draft. **Ala Abudabos**: writing – review and editing; writing – original draft. **Ibrahim A. Alhidary**: funding acquisition.

## Funding

This study was funded by The Ongoing Research Funding (ORF‐2026‐833), King Saud University, Riyadh, Saudi Arabia.

## Ethics Statement

The Committee on Animal Rights and Welfare, Faculty of Animal Husbandry & Veterinary Sciences, The University of Agriculture, Peshawar, Pakistan approved this study (FAH&VS/126 /2022).

## Conflicts of Interest

The authors declare no conflicts of interest.

## Data Availability

Data will be made available from the authors upon reasonable request.

## References

[vms370779-bib-0028] Abbas, A. , Z. Manzoor , M. Seyidov , et al. 2025. “In vivo anticoccidial, growth promoting and biochemical effects of pinus radiata extract against experimental coccidiosis in broiler chickens.” Pakistan Veterinary Journal 45, no. 3: 1383–1388. 10.29261/pakvetj/2025.268.

[vms370779-bib-0003] Aljohani, A. S. M. 2024. “Phenolics of Botanical Origin for the Control of Coccidiosis in Poultry.” Pakistan Veterinary Journal 44, no. 2: 222–228. 10.29261/pakvetj/2024.179.

[vms370779-bib-0004] Almahallawi, R. , N. Al‐Hoshani , E. Al‐Nabati , et al. 2024. “Exploring the Anticoccidial, Growth‐Promoting, Hematological, and Serological Potential Activities of *Linum usitatissimum* Essential Oil in Broiler Birds.” Pakistan Veterinary Journal 44, no. 1: 117–122. 10.29261/pakvetj/2024.137.

[vms370779-bib-0005] Alsayeqh, A. F. , and Z. A. Rao . 2023. “Nutritional Supplements for the Control of Avian Coccidiosis–A Review.” Annals of Animal Science 23, no. 4: 993–1007.

[vms370779-bib-0006] Awad, W. A. , D. Ruhnau , A. Gavrău , K. Dublecz , and M. Hess . 2022. “Comparing Effects of Natural Betaine and Betaine Hydrochloride on Gut Physiology in Broiler Chickens.” Poultry Science 101, no. 12: 102173. 10.1016/j.psj.2022.102173 PMC9573929 36228528

[vms370779-bib-0007] Batool, S. , M. A. Khan , S. Naz , et al. 2025. “Anticoccidial Effect of Bitter Apple (*Citrullus colocynthis*) Seed Powder and Organic Selenium Nanoparticles in Mitigating *Eimeria tenella* Induced Challenge in Quails.” Journal of Applied Animal Research 53, no. 1: 2526393. 10.1080/09712119.2025.2526393.

[vms370779-bib-0031] Chand, N. , S. Naz , M. Irfan , R. U. Khan , and Z. ur Rehman . 2018. “Effect of Sea Buckthorn (Hippophae Rhamnoides L.) Seed Supplementation on Egg Quality and Cholesterol of Rhode Island Red x Fayoumi Laying Hens.” Korean Journal of Food Science of Animal Resources 38: 468–475. 10.5851/kosfa.2018.38.3.468 PMC6048377 30018491

[vms370779-bib-0008] Chand, N. , P. Ali , I. A. Alhidary , et al. 2021. “Protective Effect of Grape (*Vitis vinifera*) Seed Powder and Zinc‐Glycine Complex on Growth Traits and Gut Health of Broilers Following *Eimeria tenella* Challenge.” Antibiotics 10: 186. 10.3390/antibiotics10020186. 33672923 PMC7918881

[vms370779-bib-0009] Chand, N. , S. Naz , H. Maris , R. U. Khan , S. Khan , and M. S. Qureshi . 2017. “Effect of Betaine Supplementation on the Performance and Immune Response of Heat Stressed Broilers.” Pakistan Journal of Zoology 49: 1857–1862.

[vms370779-bib-0010] Abd El‐Ghany, W. A. , and D. Babazadeh . 2022. “Betaine: A Potential Nutritional Metabolite in the Poultry Industry.” Animals 12, no. 19: 2624. 36230366 10.3390/ani12192624 PMC9559486

[vms370779-bib-0011] Ghasemi, H. A. , and N. Nari . 2020. “Effect of Supplementary Betaine on Growth Performance, Blood Biochemical Profile, and Immune Response in Heat‐Stressed Broilers Fed Different Dietary Protein Levels.” Journal of Applied Poultry Research 29, no. 2: 301–313.

[vms370779-bib-0029] Hafeez, A. , S. A. A. Shah , R. U. Khan , Q. Ullah , and S. Naz . 2020. “Effect of Diet Supplemented With Phytogenics and Protease Enzyme on Performance, Serum Biochemistry and Muscle Histomorphology in Broilers.” Journal of Applied Animal Research 48: 326–330. 10.1080/09712119.2020.1789648.

[vms370779-bib-0012] Hafeez, A. , I. Ahmad , S. Naz , et al. 2023. “Effect of Lemon (*Citrus limon* L.) Peel Powder on Oocyst Shedding, Intestinal Health, and Performance of Broilers Exposed to *Eimeria tenella* Challenge.” Animals 13: 3533. 10.3390/ani13223533. 38003150 PMC10668800

[vms370779-bib-0013] Hafeez, A. , Q. Piral , S. Naz , et al. 2023. “Ameliorative Effect of Pomegranate Peel Powder on Growth Indices, Oocyst Shedding, and Intestinal Health of Broilers Under Experimentally Induced *Coccidiosis* .” Animals 13: 3790. 10.3390/ani13243790. 38136827 PMC10740919

[vms370779-bib-0015] Ishaq, R. , N. Chand , R. U. Khan , M. Saeed , V. Laudadio , and V. Tufarelli . 2022. “Methanolic Extract of Neem (*Azadirachta indica*) Leaves Mitigates Experimentally Induced *Coccidiosis* Challenge in Japanese Quails.” Journal of Applied Animal Research 50: 498–503. 10.1080/09712119.2022.2096037.

[vms370779-bib-0016] Kar, I. , A. Mukherjee , and A. K. Patra . 2025. “Methyl Donors and Their Roles in Poultry Nutrition.” In Organic Feed Additives for Livestock, 161–173. Academic Press.

[vms370779-bib-0017] Khan, M. , N. Chand , S. Naz , and R. U. Khan . 2023a. “Dietary Tea Tree (*Melaleuca alternifolia*) Essential Oil as Alternative to Antibiotics Alleviates Experimentally Induced *Eimeria tenella* Challenge in Japanese Quails.” Animal Physiology and Animal Nutrition 107: 643–649. 10.1111/jpn.13719. 35468230

[vms370779-bib-0018] Khan, S. , N. Chand , S. Naz , et al. 2023b. “Response to Dietary Methionine and Organic Zinc in Broilers Against Coccidia Under *Eimeria tenella*‐Challenged Condition.” Livestock Science 276: 105317. 10.1016/j.livsci.2023.105317.

[vms370779-bib-0019] Nasir, J. A. , N. Chand , S. Naz , et al. 2023. “Dietary Oyster Mushroom (*Pleurotus ostreatus*) Waste Inhibits Experimentally Induced *Eimeria tenella* Challenge in Japanese Quails Model.” Animals 13: 3421. 10.3390/ani13213421. 37958176 PMC10650477

[vms370779-bib-0033] Rahman, Z. , S. Naz , R. U. Khan , and M. Tahir . 2017. “An Update on the Potential Application of L‐Carnitine in Poultry.” World's Poultry Science Journal 73: 823–830.

[vms370779-bib-0027] Rashid, S. M. , and B. H. Shnawa . 2024. “Prevalence, Morphometric, Genomic, and Histopathological Studies in Backyard Chickens Coccidiosis in Soran City, Erbil‐Iraq.” Pakistan Veterinary Journal 44, no. 2: 336–343. 10.29261/pakvetj/2024.187.

[vms370779-bib-0021] Saeed, M. , D. Babazadeh , M. Naveed , M. A. Arain , F. U. Hassan , and S. Chao . 2017. “Reconsidering Betaine as a Natural Anti‐Heat Stress Agent in Poultry Industry: A Review.” Tropical Animal Health and Production 49: 1329–1338. 28733762 10.1007/s11250-017-1355-z

[vms370779-bib-0022] Saeeda, K. , N. Chand , N. U. Khan , M. Saeed , and R. U. Khan . 2023. “Dietary Organic Zinc and Probiotic Alleviate Induced *Eimeria tenella* Infection in Japanese Quails Model of *Coccidiosis* .” Tropical Animal Health and Production 55: 37. 10.1007/s11250-022-03449-4. 36630021

[vms370779-bib-0023] Sakomura, N. K. , N. A. A. Barbosa , E. P. da Silva , F. A. Longo , I. M. Kawauchi , and J. B. K. Fernandes . 2013. “Effect of Betaine Supplementation in Diets for Broiler Chickens on Thermoneutral Environment.” Brazilian Journal of Poultry Science 8: 336–341.

[vms370779-bib-0024] Su, S. , J. Yang , L. Xu , et al. 2025. “Combined Serum Lipid Levels and Lipidomic Analysis Reveals Effects of *Eimeria maxima* and *Eimeria tenella* Infection on Lipid Metabolism in Chicken.” Veterinary Parasitology 337: 110505. 40412149 10.1016/j.vetpar.2025.110505

[vms370779-bib-0030] Ullah, F. , M. Tahir , S. Naz , N. A. Khan , and R. U. Khan . 2022. “In Vitro Efficacy and Ameliorating Effect of Moringa Oleifera on Growth, Carcass, Stress and Digestibility of Nutrients in Eschertchia Coli‐Infected Broilers.” Journal of Applied Animal Research 50: 118–124. 10.1080/09712119.2022.2039156.

[vms370779-bib-0026] Yusuf, M. S. , A. A. El Nabtiti , M. A. Hassan , and M. A. Mandour . 2018. “Supplementary Outcomes of Betaine on Economic and Productive Performance, Some Biochemical Parameters, and Lipoprotein Lipase Gene Expression in Finishing Male Broilers.” International Journal of Veterinary Science and Medicine 6, no. 2: 213–218. 30564598 10.1016/j.ijvsm.2018.11.004 PMC6286624

[vms370779-bib-0032] Zhang, P. , M. Xue , J. Gong , W. Lei , and D. Guo . 2024. “Coccidiostat Activity of Mahonia Bealei (Fort.) Leaves Extract Against Eimeria Tenella in Chickens.” Pakistan Veterinary Journal 44, no. 2: 292–297. 10.29261/pakvetj/2024.176.

